# Caregivers’ Phase-Specific Roles in Integrating Digital Technologies in Chinese Nursing Homes: Qualitative Ethnographic Study

**DOI:** 10.2196/90354

**Published:** 2026-08-07

**Authors:** Fangyuan Chang, Gubing Wang

**Affiliations:** 1 School of Design, Shanghai Jiao Tong University, Dongchuan road 800, Shanghai, Shanghai, 200240, China, 86 13167232872; 2 Tilburg University, Tilburg, North Brabant, The Netherlands

**Keywords:** nursing care, IT, system integration, caregivers, domestication theory

## Abstract

**Background:**

Digital technologies are increasingly introduced into nursing homes to address aging populations and caregiver shortages. Yet their effectiveness is often limited by rigid implementation models that overlook the role of frontline caregivers, the staff who provide hands-on daily care to residents and operate these technologies in practice. While previous studies have questioned deterministic narratives of technology adoption, less attention has been given to how frontline caregivers actively modify and reshape these systems during implementation.

**Objective:**

Drawing on domestication theory, this study examines how frontline caregivers in Chinese nursing homes negotiate, adapt, and reconfigure a newly introduced sensor-based detection system, and how their roles emerge and evolve across the full implementation process.

**Methods:**

We conducted a 6-month ethnographic study in 2 nursing homes in China. Both facilities operated under a nationally recognized welfare organization and were implementing a sensor-based detection system for fall prevention and round-the-clock monitoring. This system integrated motion sensors, pressure-sensitive mats, wearable trackers, voice-activated alerts, and a centralized data platform. Data collection combined participant observation across different implementation stages, 30 semistructured interviews with frontline caregivers selected through snowball sampling, and analysis of internal documents, including training manuals and meeting minutes. Data analysis proceeded abductively using domestication theory as a guiding framework, with field notes and interview transcripts organized chronologically across the 4 domestication phases, and coded through physical thematic mapping. A multidisciplinary team resolved cases where phase boundaries were ambiguous.

**Results:**

Frontline caregivers did not adopt the sensor system linearly or uniformly. Across the 4 domestication phases, they continuously evaluated, adjusted, and reworked the system at both individual and collective levels. In appropriation, they assessed whether the system was ethically acceptable and technically credible. In objectification, they negotiated where devices belonged, how they should appear, and how they could fit residents’ bodies and routines. In incorporation, they synchronized the system with care rhythms and absorbed the burden of system instability through hybrid manual workflows. In conversion, they translated technology use into narratives directed at families, managers, and external visitors, balancing transparency with the protection of resident dignity and privacy. Across all phases, individual judgments were progressively converted into collective norms, including documented placement standards, shift-specific monitoring protocols, and shared institutional language.

**Conclusions:**

This study highlights adaptation, rather than adoption, as the defining feature of effective technology integration in nursing homes. Caregivers’ phased and collective labor transforms a rigid monitoring system into a flexible care resource. The findings extend domestication theory beyond household contexts into institutional care settings and call for technology designs that support caregiver customization as well as policies that formally recognize caregivers’ tacit expertise.

## Introduction

The integration of digital technologies into nursing homes has emerged as a cornerstone of global strategies to address the dual crises of aging populations and chronic caregiver shortages. In China, these crises are distinctly amplified. The traditional model of family-based care has been severely strained by the 4-2-1 family structure [1], driving an unprecedented surge in demand for institutional care. To bridge this widening care gap, the Chinese government has aggressively promoted Smart Eldercare initiatives, heavily subsidizing the deployment of digital health technologies such as sensor-based detection systems across long-term care facilities [2]. Furthermore, in the Chinese sociocultural context, the shift from family-based filial piety (xiao) to institutional care creates unique expectations. Families often expect facilities to maintain a high degree of relational warmth [3], making the integration of cold, automated monitoring technologies a culturally sensitive challenge.

Yet, their implementation of these technologies often reveals profound tensions between the technical promise of these systems and the relational, ethically charged realities of caregiving [4,5]. While these technologies promise scalability, their rigid designs often clash with the fluid rhythms of care work [6], aging bodies’ heterogeneity [7], and residents’ self-efficacy [8]. In hybrid spaces such as nursing homes, bed sensors intended to ensure safety may inadvertently surveil private moments [9], and automated alerts touted as labor-saving tools frequently impose new forms of invisible work on frontline caregivers [10].

Dominant narratives in gerontechnology research often frame technology use as a binary outcome—tools are embraced for their perceived utility or rejected due to inefficacy [11]. Within this paradigm, existing studies have extensively explored caregivers’ roles, primarily focusing on their perceptions toward specific technology [12], encountered challenges [10], diverse interactions between caregivers and the selected technology [13], decision-making and learning processes [14], ethical concerns regarding safety or autonomy [15], and the meanings that caregivers assign to the selected system [16]. While providing valuable insights into the social aspects of technology integration, the body of work predominantly frames caregivers’ labor as reactive workarounds or deviations from prescribed use [17]. Consequently, institutional stakeholders often overlook caregivers’ tacit expertise, dismissing their everyday actions [18].

Grounded in science and technology studies (STS) and critical gerontology, emerging research challenges this view, revealing that caregivers rarely accept or reject systems wholesale [19]. Instead, they engage in acts of ongoing adaptations to align technologies with local values, routines, and ethical imperatives [20]. Yet, these acts of everyday innovation remain underexplored in both policy and design [21,22].

There are indeed a few studies that highlight caregivers’ active modifications and reconfigurations of these technologies in practice. For instance, Latimer and Gómez [23] position caregivers as mediators, while Chang et al [24] highlight the gaps between technology design assumptions and care realities, unraveling how caregivers could act disparately in their daily negotiations. Yet, while these studies illuminate caregivers’ individual adaptations, they largely overlook how collective norm-setting transforms localized acts into institutional policies [25]. That is, there lacks a comprehensive understanding of how caregivers, individually and collectively, play roles in transforming rigid sensor systems into flexible tools that evolve with residents’ needs throughout the whole implementation process.

To address these questions, this study uses a 6-month ethnography of 2 Chinese nursing homes implementing sensor-based detection systems. By integrating domestication theory with critical gerontology [26,27], this study aims to examine how frontline caregivers negotiate, adapt, and reconfigure these technologies, and how their specific roles emerge and evolve across the 4 domestication phases.

## Methods

### Theoretical Framework: Technology Domestication

Against deterministic views of technology adoption [28,29], domestication theory posits that a technology’s meaning and actual use emerge through active interaction with its social context [30]. Originally developed to study technologies in domestic spaces [31], the framework has increasingly been applied to institutional workplaces, including hospitals [32,33]. It conceptualizes technology integration not as a single adoption event, but as an ongoing, open-ended process across 4 distinct phases.

Appropriation refers to the initial encounter and evaluation of the technology. Unlike in household settings where users control procurement, institutional appropriation involves frontline staff making sense of a mandated system, often questioning its legitimacy and purpose. Objectification refers to the spatial and physical integration of the technology into the environment, determining where devices belong and how they align with existing spatial norms. Incorporation refers to the temporal synchronization of the technology, focusing on how devices are woven into or disrupt the everyday rhythms and routines of work. Conversion refers to the representational phase, where users mobilize the technology to construct narratives, shape institutional identity, and present their technology-mediated practices to external audiences [31].

The theory is especially potent in investigating how users relate to and interact with technology in practice over time [34]. Although current domestication studies have paid intensive attention to technology use in home settings [31,35], scholars believe the theory can also be applied in workplaces, such as institutions of higher education [36] and hospitals [32,33]. Following insights from domestication theory, the technology implementation in nursing homes should be viewed as an open and interactive process where the system is integrated into and is a part of daily structures. During the process, involved actors face a variety of changes in relation to divergent activities, spaces, times, and goals [30]. By applying this theoretical lens to nursing homes, we shift the analytic focus from whether a technology is accepted or rejected to the complex, phase-specific labor caregivers perform to make the system workable within the ethical and practical realities of daily care.

### Research Contexts: Caregivers and Sensor-Based Detection System

In Chinese public nursing homes, caregivers (often referred to as Hu Gong) play a central role in residents’ daily care [37]. Unlike registered nurses (often referred to as Hu Shi), who are autonomous professionals working within well-established clinical standards and interdisciplinary care structures, caregivers typically enter the profession through short-term training programs without formal higher education in health care [37]. Despite their limited formal qualifications, caregivers are indispensable in managing residents’ daily routines, personal hygiene, and emotional well-being. They are responsible for hands-on care tasks under the supervision of registered nurses in nursing homes. Hence, caregivers are typically the ones working on the frontline, using the newly introduced technologies in everyday care services [38].

The sensor-based remote monitoring system used in this study was developed and implemented as part of a national innovation initiative aimed at advancing the digital transformation of older adult care facilities across the country. Among various technological solutions piloted under this initiative—such as robotic assistants, AI-driven diagnostic tools, and telemedicine platforms—the sensor-based detection system stood out for its features of noninvasive monitoring, real-time responsiveness, and high cost efficiency [39].

The system’s architecture was purpose-built to address critical challenges in older adult care settings, particularly fall prevention and round-the-clock monitoring [40]. At its core, the system integrated a multimodal sensor network, including infrared motion detectors, pressure-sensitive mats, wearable mobility trackers, and bed-exit alarms, all synchronized through a centralized data platform. Complementing these components were microphones with voice-activated alerts, enabling caregivers to receive audio-based notifications—such as calls for assistance or unusual vocal patterns—while also facilitating 2-way communication between residents and caregivers. Touchscreen tablets served as interactive interfaces, displaying real-time alerts and allowing caregivers to adjust settings or review data logs. The system’s AI algorithms were trained to distinguish between routine activities and potential risks, such as sudden falls or prolonged immobility, triggering tiered alerts ranging from low-priority notifications for irregular movement patterns to emergency signals for critical incidents. Real-time alerts were complemented by data logging capabilities, which generated metrics such as fall rates, mobility trends, and AI-driven risk scores to inform care decisions.

The implementation program began with vendor-led training sessions. The system remained under iterative development throughout this study, with an in-house engineering team optimizing device aesthetics, alert sound profiles, and algorithmic accuracy based on real-world feedback. This system was chosen as the empirical focus due to its role as a nationally prioritized innovation. Its multifaceted design—combining motion sensors, fall detection, voice-activated alerts, and adaptive wearables—provided a holistic lens to study how interconnected technologies interact with caregiving’s fluid realities. The system’s ongoing development phase allowed observation of real-time negotiations between technological promises and care practices.

### Qualitative Approach and Research Paradigm

This study adopts an interpretivist, ethnographic approach [41] and was reported in accordance with the Standards for Reporting Qualitative Research guidelines (the completed checklist is provided in Checklist 1). Ethnography was selected because it enables sustained, contextually embedded observation of everyday practices. Domestication theory was adopted as the guiding analytical framework before data collection. Data collection and analysis proceeded abductively, moving iteratively between empirical observations and theoretical concepts to allow patterns to emerge from the data while remaining theoretically informed.

### Researcher Characteristics and Reflexivity

The first author, trained in STS with approximately ten years of ethnographic fieldwork experience, conducted all participant observations. Before entering the field, the research team held an interpretivist assumption that caregivers’ relationships with the sensor system would vary across domestication phases and that their roles would be active rather than merely reactive. To manage these presuppositions, the first author adopted a stance of empathetic listening and nonjudgmental inquiry throughout the fieldwork, deliberately withholding analytic judgments during early observations to allow unexpected patterns to surface. The second author, while unable to maintain full-time field presence due to scheduling constraints, participated in selected on-site activities, monitored real-time updates via the institutional implementation group chat, and engaged in regular analytical discussions with the first author. Instances of analytical inconsistency were brought to a broader multidisciplinary team—comprising researchers with backgrounds in STS, social science, and interaction design—for structured discussion and resolution during team meetings.

### Study Setting and Case Selection

This paper presents data from a 6‐month ethnographic study conducted in 2 middle-sized nursing homes—representative of the most common size for such facilities in China. One nursing home is located in an urban area, while the other is situated in a rural area (Multimedia Appendix 1). Although the geographical contexts differ, both nursing homes share similar organizational structures, staffing patterns, and technological infrastructures. They are engaged in ongoing efforts to enhance technology integration in daily care, using the same sensor-based detection systems and related sensing devices.

To select suitable research sites, we collaborated closely with an innovative technology company specializing in sensing technology for nursing care. Through the company leader, we obtained contact information for 14 nursing homes that had recently adopted or planned to adopt these technologies. However, several nursing homes declined participation due to concerns such as privacy issues and operational inconvenience. Among the remaining 8 facilities, 3 facilities were excluded because they already had established technological infrastructures and were focused on system renovations. Of the remaining 5 facilities, 2 were selected due to their similarities in features such as organizational structures and staffing patterns.

Both nursing homes operated under the auspices of a nationally recognized welfare organization, representing among the highest-tier older adult care institutions in their respective regions. As nationally recognized demonstration facilities, both nursing homes also regularly hosted visits from managers and practitioners from other institutions and were expected to produce public-facing materials documenting their care practices. As reported by institutional managers during initial site negotiations, caregivers at these facilities were required to hold a college degree or above, possess relevant vocational qualifications, and have a minimum of 2 years of professional experience. The two nursing homes served older adults with varying levels of care needs, including residents with mobility limitations, recurrent fall risk, and cognitive impairment. Caregivers’ accounts and field observations indicated that residents’ conditions ranged from relatively stable physical dependency (eg, requiring moderate assistance with bathing, dressing, or mobility) to more complex situations involving Parkinsonian tremors and dementia-related confusion. The majority of the monitored population required continuous daily living assistance, which structured the caregivers’ routines.

### Data Collection

#### Participant Observation

The first author conducted participant observation in both nursing homes. The observation periods were designed to correspond with distinct stages of the system implementation timeline: the first period captured caregivers’ initial encounters with the technology during early deployment; the second documented emerging routines and adaptations as the system became more embedded in daily workflows; and the third traced the consolidation and institutionalization of caregiving practices around the technology. The intervals between observation periods maintained active field contact: the first author sustained informal communication with caregivers and received real-time institutional updates via a dedicated implementation group chat shared among caregivers, managers, and the engineering team. This design enabled longitudinal tracking of domestication as a dynamic, evolving process rather than a static snapshot. Immersion in the field involved:

- Regarding daily engagement, the first author focused on shadowing frontline caregivers during their shifts, observing routines including care rounds, medication administration, and resident interactions. Special attention was given to moments when caregivers encountered technological tools during scheduled training sessions and in day-to-day use.
- Regarding contextual immersion, this study included participation in staff meetings, informal debriefings during breaks, and other moments of interaction in communal spaces such as break rooms and staff lounges. Field notes were recorded, capturing detailed descriptions of how caregivers appropriated, reconfigured, and integrated technology within their care routines. These notes not only detailed observable practices but also documented the ambient conversations and subtle negotiations around the use and meaning of technological tools.
- Regarding participatory observations, at various points throughout this study, the researcher undertook mini-immersions lasting several days to better understand the temporal dynamics of technology integration. These episodes allowed for a deep dive into the evolution of practices over time.

#### Semistructured Interviews

To complement the observational data, a total of 30 semistructured interviews were conducted with frontline caregivers. Participants were identified through snowball sampling, beginning with caregivers who had established rapport with the first author during participant observation and who expressed willingness to share their experiences in depth. Initial participants were invited to recommend colleagues who had been involved in different aspects of the system implementation, progressively expanding the sample to include caregivers across varied shift patterns, years of professional experience, and stages of technology engagement. Selection decisions were made jointly by the first and second authors. All participants were frontline caregivers with direct, daily interaction with the sensor system; registered nurses and managerial staff were excluded as their roles in system implementation differed substantially. Fifteen participants were recruited from each institution (Table 1).

**Table 1.** An overview of participant characteristics.

ID	Sex	Age (years)	Work experience (years)	Institution
P01	Female	24	2	Urban nursing home
P02	Female	27	4	Urban nursing home
P03	Male	29	5	Urban nursing home
P04	Female	31	6	Urban nursing home
P05	Female	33	8	Urban nursing home
P06	Female	35	9	Urban nursing home
P07	Male	37	6	Urban nursing home
P08	Female	39	2	Urban nursing home
P09	Female	41	14	Urban nursing home
P10	Female	43	12	Urban nursing home
P11	Male	45	8	Urban nursing home
P12	Female	47	8	Urban nursing home
P13	Female	49	11	Urban nursing home
P14	Female	52	20	Urban nursing home
P15	Male	55	12	Urban nursing home
P16	Female	23	4	Rural nursing home
P17	Female	26	3	Rural nursing home
P18	Female	28	4	Rural nursing home
P19	Male	30	6	Rural nursing home
P20	Female	32	7	Rural nursing home
P21	Female	34	8	Rural nursing home
P22	Male	36	10	Rural nursing home
P23	Female	38	12	Rural nursing home
P24	Female	40	13	Rural nursing home
P25	Female	42	15	Rural nursing home
P26	Female	44	17	Rural nursing home
P27	Male	46	18	Rural nursing home
P28	Female	48	20	Rural nursing home
P29	Female	50	14	Rural nursing home
P30	Female	53	21	Rural nursing home

Given this study’s focus on role emergence across a full implementation timeline, the sample size was expanded beyond what is typical in single-context ethnographic studies to ensure adequate representation across different stages of the domestication process. The full interview guide is provided in Multimedia Appendix 2. The interviews aimed to capture:

- Domestication experiences in which guided by the 4 phases of domestication theory (appropriation, objectification, incorporation, and conversion), interview protocols probed into the roles that caregivers play at each stage. Questions were designed to elicit detailed narratives about how these devices were first encountered, how they were subsequently integrated into daily routines, and how their use was negotiated, reconfigured, or even resisted in practice.
- Attitudes and perceptions in which each interview also explored the caregivers’ personal attitudes toward the introduction and adaptation of technological solutions. For instance, interviewees were asked how they made sense of new technology, how they assigned meaning to devices within the context of care, how they felt about the adaptations of these tools in their workflow, and how they viewed the benefits or drawbacks of technology use with peers and residents.

All interviews were audio-recorded with participants’ consent and transcribed verbatim using Tencent Meeting’s automated transcription function, followed by manual verification by both authors to ensure accuracy. Transcripts were produced in Chinese, the language in which all the interviews were conducted. Relevant excerpts were subsequently translated into English by the first and second authors collaboratively, following an edited verbatim approach: the original meaning was preserved while false starts and redundant utterances were removed for clarity.

#### Document Analysis

In addition to participant observation and interviews, this study incorporated a detailed analysis of internal documents such as:

- Training manuals and usage guidelines for which these documents outlined the official protocols for technological integration and served as a benchmark against which actual practices could be compared.
- Meeting minutes and internal communications in which records from staff meetings and technology implementation briefings provided context on the formal narratives surrounding technology use and the intended roles of caregivers in the domestication process.

These documents helped triangulate the observational and interview data, offering a multilayered understanding of both the prescribed and emergent aspects of technology integration.

### Data Analysis

Data analysis proceeded abductively and iteratively, maintaining a continuous dialogue between empirical observations and domestication theory. As the 4 domestication phases corresponded naturally to the system implementation timeline, field notes and interview transcripts were first organized chronologically and assigned to phases based on the stage of implementation during which each observation or interview occurred. Within each phase, open coding was conducted manually by the first and second authors using a physical thematic mapping approach: data-derived codes were written on adhesive notes and organized on a whiteboard, allowing caregiver roles to emerge visually from the data. Most codes were assigned to phases with high confidence. Where phase boundaries were ambiguous, cases were brought to the broader multidisciplinary research team for structured discussion during team meetings, with final assignments reached through consensus. To preserve the cultural and contextual nuances of the narratives, all qualitative coding and data analyses were conducted directly on the original Chinese verbatim transcripts rather than on the translated versions. Translated excerpts were used only for manuscript presentation.

The remaining elements of the analysis unfolded in several stages:

- Regarding theoretical sensitization, with domestication theory serving as a guiding framework, the analysis then focused on mapping practices within the 4 phases of technology domestication (ie, appropriation, objectification, incorporation, and conversion), examining how caregivers’ roles emerged and evolved within each phase.
- Regarding iterative refinement, the coding process was iterative. As patterns emerged, higher-order codes were developed to capture the roles that caregivers play in transitioning between these phases. Particular attention was paid to the subtle and often tacit ways in which caregivers negotiated the intended functions of technology with the practical demands of care work. The analysis sought similarities in these adaptive strategies across both nursing homes, revealing common threads in the domestication process.
- Regarding memo writing and synthesis, throughout the analysis, detailed memos were written to document evolving insights and to reflect on discrepancies between the formal expectations outlined in internal documents and the actual practices observed on the ground. Special attention was paid to moments when caregivers expressed frustrations, creative workarounds, or subtle forms of resistance to the prescribed use of technology.

### Techniques to Enhance Trustworthiness

Several techniques were used to enhance the trustworthiness of the findings. Prolonged engagement in the field across 6 months, combined with continuous contact during interobservation intervals, allowed the first author to develop deep familiarity with the care environment and to observe practices across the full system implementation cycle [42]. Triangulation was achieved through the integration of 3 data sources (ie, participant observation, semistructured interviews, and institutional documents), enabling cross-verification of emerging patterns. Analytical consistency was supported through regular discussions between the first and second authors, with ambiguous cases escalated to a multidisciplinary team for consensus-based resolution. Reflexivity was maintained through analytical memo writing, in which the first author documented evolving interpretations and monitored the influence of prior assumptions on data analysis. Translated excerpts were reviewed by both authors to ensure fidelity to participants’ original intended meanings. Member checking was conducted at 2 levels: informally, through ongoing conversations with caregivers during fieldwork in which emerging interpretations were shared and refined; and formally, through a presentation of findings to all participating caregivers and institutional managers upon completion of analysis, providing an opportunity for participants to verify, contest, or elaborate on the research conclusions.

### Ethical Considerations

This study was approved by the Ethics Committee of Shanghai Jiao Tong University (H20240125P-R1). Informed consent was obtained from all participants before data collection, with assurances of confidentiality and anonymity. All data were anonymized through the assignment of coded identifiers, and no personally identifying information was retained in transcripts or field notes. No financial compensation was provided to participants.

## Results

### Overview

In the 2 Chinese nursing homes, caregivers did not adopt the sensor-based detection system linearly or uniformly. Instead, they continuously evaluated, adjusted, and reworked it so that it could fit the moral, spatial, temporal, and organizational realities of care. Across the 4 domestication phases, the system was gradually transformed from an externally introduced monitoring tool into a resource that caregivers could make usable, defensible, and locally meaningful. Table 2 provides an overview of the 16 roles identified across the 4 phases.

Each phase corresponds to a distinct stage of the system implementation timeline. Roles at the individual level reflect caregivers’ direct, situated engagements with the technology and with residents. Roles at the collective level reflect how individual adaptations were progressively codified into shared norms, protocols, and institutional identity.

**Table 2.** An overview of caregivers’ roles across phases.

Phase and level	Overarching roles	Micropractices
Appropriation		
Individual	System evaluators	Ethical auditors Independent validators
Collective	Consensus builders	Knowledge aggregators Collective sense makers
Objectification		
Individual	Context reconfigurers	Spatial mediators Interface negotiators
Collective	Norm setters	Spatial standardizers Cultural weavers
Incorporation		
Individual	Workflow adapters	Temporal integrators Glitch navigators
Collective	Protocol synchronizers	Shift harmonizers Protocol innovators
Conversion		
Individual	Data translators	Human-centered storytellers Privacy guardians
Collective	Value stewards	Institutional ambassadors Ethos stewards

### Appropriation: Assessing Legitimacy and Building Shared Meaning

In the appropriation phase, caregivers’ first question was not how to use the system, but whether it should be trusted. They evaluated it as both a technical device and a moral intervention in residents’ lives.

At the individual level, caregivers acted as system evaluators, scrutinizing the system’s legitimacy on both moral and technical fronts. On the one hand, they functioned as ethical auditors, considering whether microphones, alerts, and monitoring functions respected residents’ dignity and privacy. During training, P05 challenged the bathroom audio feature and questioned whether a private space should contain a microphone. P12 described turning off a voice-activated alert after noticing that a resident felt uneasy around the device. She said she would rather accept some risk than make the resident uncomfortable, though she added that she “worried someone would notice and ask” (P12, female, 8 y, urban). These decisions were rarely recorded formally, but they shaped what counted as acceptable use in practice. On the other hand, because confidence in the system varied widely across staff, some caregivers worked within this evaluation role as independent validators. Rather than accepting vendor claims at face value, they checked whether the system actually behaved as promised. P09 searched online nursing forums after hearing claims of zero false alarms, then tried to test fall detection herself by dropping a weighted pillow from different heights. She acknowledged the limits of this approach: “I’m not an engineer. I just wanted to see for myself” (P09, female, 14 y, urban). This kind of informal verification helped caregivers decide which functions were usable and which were reliable to be trusted. During observation, such informal technical verification was more often initiated by experienced staff, whereas junior caregivers tended to adopt these practices through observation and imitation.

At the collective level, caregivers transitioned into consensus builders**,** pooling dispersed observations to forge shared practice and values. Some staff acted as knowledge aggregators. P14 described how staff began writing down recurring problems and fixes in a shared spreadsheet, while P22 said that a living document had become the place where caregivers recorded their coping strategies: “Sometimes we don’t know if the fix is right, but at least the next person knows what we tried” (P22, male, 10 y, rural). These records became an informal knowledge base for the team and a way for new staff to learn how the system actually behaved in context. Simultaneously, caregivers operated as collective sense makers, negotiating what the system meant in relation to care values. In staff meetings, caregivers spontaneously debated whether algorithmic risk scores should influence care planning. P11 objected to letting a computer shape whether a resident should receive fewer visits. The team ultimately decided not to include system recommendations in care logs. Such debates over where to draw the line between system output and care judgment happened repeatedly, shaping what the team counted as legitimate use.

### Objectification: Negotiating Place, Appearance, and Bodily Fit

In the objectification phase, caregivers worked out where the system belonged in the physical and social environment of the nursing home. This was not only a matter of installation but a process of deciding how the system should appear, where it could be seen, and how it might fit residents’ bodies and routines.

At the individual level, caregivers acted as context reconfigurers, adjusting physical hardware and interfaces to achieve situational compatibility. As spatial mediators, they repositioned devices to reduce discomfort while preserving function. P10 described a resident who covered a sensor with a scarf and emphasized the bedroom as a private place. She moved the sensor to a ceiling corner and softened its appearance. During the process, she “had to ask the engineer twice” in case the changes affected the system performance (P10, female, 12 y, urban). In another case, a caregiver repurposed a bed-exit alarm’s receiver into a more discreet decoration form after a resident said the wall-mounted unit “made the room feel like a laboratory” (P19, male, 6 y, rural). In addition to repositioning devices, caregivers also adapted the system to residents’ bodies. P25 spent 2 nights calibrating pressure-sensitive mats for a resident with Parkinson disease, whose tremors triggered constant false alarms: “The first night I gave up around 11 PM I tried again the next night” (P25, female, 15 y, rural). She acknowledged that the calibration was provisional, claiming that if the resident’s medication changed, the work would have to be redone. P20 lowered motion sensitivity after a resident’s nighttime restlessness repeatedly triggered false alarms: “It worked for some, didn’t work for others” (P20, female, 7 y, rural). These calibrations were ongoing negotiations between technical settings and bodily variation. For residents with cognitive impairment, caregivers worked as interface negotiators. P13 described a resident with dementia who reacted to the blinking light on the wall-mounted motion sensor as a sign of malfunction: “She’d point at it and say, 'It’s broken again!' and try to unplug it. Eventually I just stopped explaining” (P13, female, 11 y, urban). She placed a piece of frosted tape over the light, after which the resident walked past the sensor without comment. This was not simply a visual tweak. It was a way of making the device legible within the resident’s perceptual world.

At the collective level, caregivers operated as norm setters, stabilizing individual reconfigurations into shared institutional aesthetics and rules. Many acted as spatial standardizers, codifying placement practices across shifts and teams. Caregivers developed shared understandings about where devices did and did not belong. For instance, sensors should not face a resident’s bed directly, and installation requires at least a verbal agreement from the resident. When family gatherings began taking place in renovated lounges, caregivers collectively moved sensors away from those areas so that visitors would not feel watched. Beyond placement norms, caregivers also acted as cultural weavers, working together to change how technology felt to residents. They began decorating fall-alert pendants with beads, ribbons, and small charms, turning devices into personalized objects such as jade trinkets. Management initially dismissed this as frivolous, but the practice reduced device abandonment over this study’s period. Building on the same logic, caregivers later persuaded engineers to replace harsh alarm tones with bird-song variations. Not every resident participated, and those who declined were always respected. These actions show that objectification was not passive placement but a process of local recontextualization.

### Incorporation: Synchronizing the System With Care Rhythms

In the incorporation phase, caregivers focused on how the system fit into the temporal structure of care. The central problem was not whether the system could function technically, but whether it could function without disrupting the daily rhythms of staff and residents.

At the individual level, caregivers acted as workflow adapters, managing the microtempo of care and absorbing operational friction. Operating as temporal integrators, P02 adjusted alert intervals to avoid repeated interruptions during busy periods. She lengthened the motion sensor alerts to every 30 minutes during breakfast and bathing when staff were already present, then restored the default 15-minute interval during quieter afternoon hours. For residents who wandered at night, caregivers often suspended bed-exit alerts and substituted direct checks. P24 explained that a resident’s nighttime pacing was not necessarily something to suppress. It was part of how she coped. In these cases, caregivers did not simply follow the system’s timing. They redefined safety around the actual rhythm of the person. Furthermore, when the system failed, caregivers became glitch navigators. False alarms were frequent during the initial installation of the detection system. P17 and a colleague split the work between manual room checks and manual logging to ensure resident safety. After 2 weeks of stable system usage following iterative adjustments to minimize false alarms, they still kept the strategies: “The system worked these days. But we couldn’t just trust it…what if a real fall happened whereas there was no alarm?” (P17, female, 3 y, rural). These hybrid work patterns show that caregivers absorbed much of the burden created by system instability, often maintaining parallel manual checks even after the system became more stable.

At the collective level, caregivers operated as protocol synchronizers, aligning operational schedules across the organization. Acting as shift harmonizers, they coordinated goals across teams. P15 described how day staff and night staff initially prioritized different goals. One group focused on fall prevention, while the other focused on protecting sleep. “We argued for weeks before we agreed on shift-specific profiles” (P15, male, 12 y, urban). The team also muted noncritical alerts during family visits so that alarms would not interrupt relational moments. Some staff initially resisted these blackouts, worried about safety gaps, but the practice held after a few months of trial. They later created a laminated decision flowchart, which P03 called “our peace treaty between shifts” (P03, male, 5 y, urban). Caregivers also acted as protocol innovators. In rehabilitation areas, motion sensors repeatedly produced false alarms during therapy sessions because deliberate stretching movements resembled falls. P23 described how the team responded by muting nonurgent alerts during therapy hours and checking cameras before escalating, and by linking therapy schedules to sensitivity settings so that the system could reflect the actual organization of care work. Although caregivers noted that new sources of alarm continued to surface as residents’ needs shifted, the protocol proved efficient in reducing false alarms over time. Taken together, these practices show that incorporation was not merely a matter of individual adjustment. It also required negotiated coordination across teams and the ongoing translation of recurrent frictions into shared operational routines.

Across these incorporation practices, caregivers’ room for maneuver was not distributed evenly. Junior staff, such as P17, were more likely to absorb system instability through manual checking, logging, and other labor-intensive workarounds, whereas more experienced caregivers were better positioned to escalate recurring frictions into shift-wide procedural adjustments. At the same time, once such responses were formalized into shared routines, some of these experience-based differences became less visible in day-to-day practice, as junior staff adopted protocols initiated by more senior colleagues.

### Conversion: Translating Use Into Narrative and Institutional Identity

In the conversion phase, caregivers transformed local technology use into narratives for multiple, sometimes conflicting, audiences: family members concerned about a relative’s safety, managers focused on performance metrics, and professional visitors expecting evidence of the facility’s digital care model.

At the individual level, caregivers serve as data translators, contextualizing and filtering digital outputs to protect residents. As human-centered storytellers in ordinary conversations with families, caregivers rarely raised system data on their own. However, when a family member asked directly, or when data had to be presented in a formal setting, caregivers worked to give the numbers a recognizable shape. P14 described how she handled a daughter’s question about her father’s progress (P14, female, 20 y, urban). Rather than reporting the alert count alone, she paired it with the timeline of his physiotherapy: “as he gained confidence walking, the alerts dropped.” P08 had learned the importance of this framing the difficult way. After a family misinterpreted an alert log as evidence that staff was missing residents’ calls, she revised how she introduced data: “Now I preface reports with context...like explaining that a spike in alerts often means we’re catching things early, not missing them. It took me a while to realize this context must be clarified when presenting the data” (P08, female, 2 y, urban). This kind of storytelling was rarely scripted in advance. It required judgment about the listener and a clear understanding of associated contexts. Minor variations also emerged in how caregivers performed this data translator role. In our observations, professional seniority occasionally shaped how fluently caregivers contextualized system data for families: veteran staff such as P14 tended to do so more readily, whereas junior staff such as P08 often described learning through earlier misinterpretations and subsequent adjustments. Caregivers also served as privacy guardians. In clinical reports reviewed by physicians, full data records were retained, but when preparing summary reports for management, caregivers stripped identifying details, presenting unit-level trends. P29 explained the logic: management needed to know if fall risk had fallen across the unit, but not which resident was associated with which alert (P29, female, 14 y, rural). A similar logic applied to the materials prepared for external audiences. Caregivers reviewed draft documents and removed case-level details that could identify individual residents, replacing them with anonymized trends. Over time, the result was a tiered practice in which the same underlying data was made differently visible to different audiences.

At the collective level, caregivers worked as value stewards, governing how technology-mediated care was conceptualized and projected institutionally. Acting as institutional ambassadors, they shaped how the nursing home as a whole spoke about the system. P15 explained that new staff were trained to describe the system as helping the team notice small changes early, rather than as a monitoring tool (P15, male, 12 y, urban). The shift was not absolute in the beginning. Older terms continued to surface in informal conversation, and not all staff adopted the new language consistently. However, the internal documents shifted over time, where phrases such as resident-centered alerts replaced earlier framings. The material environment was also adapted to support this framing. Devices were gradually covered in different materials that blended them into the décor. P26 noted that the visual softening changed how visitors and residents related to the devices in the room (P26, female, 17 y, rural). The same tension between transparency and dignity surfaced when caregivers prepared materials for professional visitors, where caregivers worked as ethos stewards. On one side, demonstration sites were expected to document measurable outcomes. On the other hand, caregivers were committed to not reducing residents to data points. During one drafting session, caregivers debated whether to lead with a graph of decreased fall rates. P11 raised the concern that doing so risked treating residents as problems to be fixed (P11, male, 8 y, urban). The debate ran across 2 meetings before the team agreed on a compromise: the graph would appear, but it would be paired with anonymized vignettes describing residents’ return to ordinary activities. Before finalizing the document, the team consulted with the residents’ families. One daughter asked that her mother’s story focus on regained independence rather than sensor metrics. The final version opened with a caregiver’s reflection: “We don’t chase perfect data. We chase mornings where Uncle Li feels safe enough to make his own tea” (P10, female, 12 y, urban).

## Discussion

### Principal Findings

Our study reveals a dynamic, nonlinear process through which caregivers domesticate remote monitoring technologies in nursing homes, challenging the linear adoption narratives that often shape discussions of health care technology [11]. Across the 4 phases of domestication, caregivers were not simply users of the system but active participants in its ongoing adjustment, reinterpretation, and stabilization. Their work moved across individual and collective levels, as localized adaptations were gradually translated into shared routines, protocols, and representational practices.

### Caregivers as Co-Designers in Technology Reconfigurations

The findings extend dominant models of health care technology adoption, such as the technology acceptance model [43], which conceptualizes adoption as an outcome driven by perceived usefulness and ease of use. Rather than accepting or rejecting technologies, caregivers actively reconfigure them through situated practices that combine ethical reasoning, spatial adjustment, and workflow adaptation. For example, within their core capacity as system evaluators, caregivers engage as ethical auditors to interrogate system features against care values, often modifying or disabling functions that conflict with resident dignity. Similar observations have been noted in prior work on technology mediation in care [21]. Yet, our findings further show that such actions were not isolated acts of resistance, but part of an ongoing effort to realign system functions with the practical and moral demands of care. Likewise, as collective norm setters, caregivers operate as cultural weavers to transform devices into socially meaningful artifacts (eg, decorated pendants), echoing prior discussions on stigma and assistive technologies [44]. This case suggests how aesthetic and symbolic modifications are embedded in everyday care practices.

Spatial and interface-level adaptations further illustrate caregivers’ co-design agency. Consistent with critiques of unobtrusive computing in gerontechnology [45], caregivers in our study do not necessarily minimize visibility. Instead, they recontextualize devices to align with residents’ cognitive and emotional needs (eg, embedding interfaces into familiar forms). These practices resonate with prior STS accounts of users reshaping technologies in use [18], but highlight a stronger emphasis on relational compatibility in institutional care settings.

Taken together, these findings suggest that domestication in nursing homes is not well captured as a simple matter of acceptance or rejection. Instead, it involves continuous reconfiguration, through which caregivers iteratively align technical functions with care values, resident needs, and the practical organization of work. In this sense, our findings complement acceptance-oriented models such as the technology acceptance model by foregrounding forms of collective, situated, and ongoing work that those models do not readily capture.

### Temporal Fluidity and the Burden of Invisible Labor

Our findings also foreground the temporal dimension of technology integration, aligning with the notion of the logic of care by Mol [46], where care practices are shaped by responsiveness to lived conditions rather than standardized efficiency. Caregivers act as individual workflow adapters, dynamically embodying temporal integrators who adjust alert rhythms and system sensitivities to match residents’ daily routines and bodily rhythms. These adaptations challenge efficiency-driven system logics that assume uniform patterns of behavior [47]. At the same time, these adjustments rely on substantial invisible labor. Micropractices such as troubleshooting system failures and developing shared protocols demonstrate how caregivers continuously bridge gaps between technological expectations and real-world contingencies. This aligns with the concept of infrastructural work by Gui and Chen [48], where the maintenance and repair of systems remain largely unrecognized despite being essential to their functioning.

Our analysis also suggests that this invisible labor was not distributed evenly. In particular, differences in professional seniority and accumulated institutional experience shaped caregivers’ room for maneuver during incorporation: junior caregivers more often absorbed technological friction through manual workarounds, whereas more experienced staff were better positioned to escalate recurring problems into shift-wide protocols. Once such responses were formalized into shared routines, however, some of these experience-based differences became less visible in day-to-day practice.

Our data also reveal tensions within this process. For instance, the need to maintain temporal flexibility during system failures can conflict with efforts to standardize workflows across shifts [49]. These tensions illustrate a broader paradox in digital care systems: while technologies aim to enhance efficiency and standardization, their effective use often depends on situated, adaptive, and labor-intensive practices.

By making these forms of labor visible, this study contributes to ongoing discussions about the hidden costs of digital health technologies [17,50], suggesting that successful implementation depends not only on system design but also on the sustained and often unacknowledged work of frontline caregivers.

### From Individual Scrutiny to Institutional Codification

Our findings extend domestication theory [31] beyond household contexts by demonstrating how technology integration in institutional settings involves collective processes of negotiation and stabilization. While domestication has traditionally emphasized individual meaning-making, our study shows how caregivers’ adaptations are progressively codified into shared norms, protocols, and organizational practices.

For example, individual acts of ethical resistance (eg, disabling intrusive features) are transformed into collective decisions through team discussions, while localized troubleshooting efforts evolve into shared knowledge repositories. These processes align with the concept of epistemic practices by Cunningham and Kelly [51], where knowledge is collaboratively constructed through situated problem-solving. Through their roles as individual data translators and collective value stewards, caregivers actively shaped how technology-mediated care was described, justified, and made legible to different audiences within and beyond the institution. In doing so, they often prioritized narratives of resident dignity and relational care over the standalone presentation of quantitative metrics.

These narrative practices are deeply rooted in the Chinese sociocultural context, where expectations of filial piety (xiao) place strong emphasis on relational warmth in older adult care [3]. By filtering rigid alert metrics into human-centered stories of regained independence, caregivers sought to reassure families that institutional care remained a space of human touch rather than sterile surveillance, thereby making digital monitoring more compatible with culturally grounded expectations of care.

Taken together, these findings shift the analytic focus from individual adaptation to institutional codification. In nursing homes, domestication was not only about whether caregivers accepted a technology, but about how frontline staff collectively stabilized it through negotiation, workaround, selective visibility, and protocol-making. This extends domestication theory beyond household settings by showing that, in institutional care, technology integration hinges less on user-controlled acquisition and more on collaborative adjustment, negotiation, and formalization within ongoing care work. Practically, the findings also caution against top-down digitization agendas that treat implementation as a matter of deployment alone. Remote monitoring systems became workable not because they were simply introduced into care settings, but because caregivers continuously reshaped them to align with resident dignity, relational expectations, and the temporal rhythms of care.

### Limitations and Future Work

This study has several limitations. First, the ethnographic focus on 2 nursing homes limits the generalizability of findings. The forms of caregiver agency observed here may vary in settings with different resource levels, organizational structures, or cultural norms. Future studies could examine how these dynamics unfold in more resource-constrained environments or in systems with stricter hierarchical control.

Second, although residents indirectly influenced the domestication process (eg, through acceptance, resistance, or feedback), their perspectives were not systematically captured. Given the relational nature of care, future research should more explicitly include residents as active participants in technology domestication.

Last, this study is situated in a Chinese context, where collectivist norms and institutional arrangements may shape how technologies are negotiated and integrated. Comparative studies across cultural contexts would help clarify which aspects of caregiver roles are context-specific and which are more generalizable.

### Conclusions

This study set out to examine how frontline caregivers in Chinese nursing homes engage with a newly introduced sensor-based detection system across the full implementation process. Rather than uniformly adopting the system, caregivers continuously evaluated, adjusted, and reworked it to fit the moral, spatial, temporal, and organizational realities of their work. The 16 roles identified across the 4 domestication phases suggest that technology integration in nursing homes is less a process of adoption than one of ongoing adaptation, shaped by both individual judgment and collective negotiation.

These findings carry several practical implications. For technology designers, they suggest that systems intended for institutional care benefit from being open-ended and adjustable, rather than optimized for a single use pattern. Alert intervals, sensitivity thresholds, and data visibility are not fixed technical parameters but practical decisions that frontline caregivers are better positioned to make in context. For those responsible for implementation and training, the findings suggest that one-off vendor-led instruction captures only the beginning of the process. Caregivers’ expertise continues to develop as they live with the technology, and institutional structures that support the sharing of that expertise appear to matter. For policy, there may be value in formally recognizing the kind of labor this study describes and the narrative work that caregivers routinely perform, but that rarely appears in institutional metrics.

By identifying phase-specific roles and tracing how individual adaptations evolve into collective practices, this study contributes to both domestication theory and research on digital health implementation. The findings suggest that effective technology integration depends not only on system design but also on recognizing and supporting caregivers’ ongoing, situated work.

These insights have implications for the design of flexible systems, the development of training programs that acknowledge caregiver expertise, and policy frameworks that move beyond adoption metrics to account for the realities of care practice.

## Supplementary material

10.2196/90354 Multimedia Appendix 1 Setting profiles.

10.2196/90354 Multimedia Appendix 2 Interview schedule.

10.2196/90354 Checklist 1 SRQR Checklist.

## Abbreviations

STS: science and technology studies

## References

[1] Jiang Q, Sánchez-Barricarte JJ (2011). The 4-2-1 family structure in China: a survival analysis based on life tables. Eur J Ageing.

[2] Chen H, Hagedorn A, An N (2023). The development of smart eldercare in China. Lancet Reg Health West Pac.

[3] Zhan HJ, Feng Z, Chen Z, Feng X (2011). The role of the family in institutional long‐term care: cultural management of filial piety in China. Int J Soc Welfare.

[4] Conte G, Arrigoni C, Magon A, Stievano A, Caruso R (2023). Embracing digital and technological solutions in nursing: a scoping review and conceptual framework. Int J Med Inf.

[5] Huter K, Krick T, Domhoff D, Seibert K, Wolf-Ostermann K, Rothgang H (2020). Effectiveness of digital technologies to support nursing care: results of a scoping review. J Multidiscip Healthcare.

[6] Muusse C, Kroon H, Mulder CL, Pols J (2021). Frying eggs or making a treatment plan? Frictions between different modes of caring in a community mental health team. Sociol Health Illness.

[7] Taipale S, Oinas T, Karhinen J (2021). Heterogeneity of traditional and digital media use among older adults: a six-country comparison. Technol Soc.

[8] Park HK, Chung J, Ha J (2023). Acceptance of technology related to healthcare among older Korean adults in rural areas: a mixed-method study. Technol Soc.

[9] de Zambotti M, Goldstein C, Cook J (2024). State of the science and recommendations for using wearable technology in sleep and circadian research. SLEEP.

[10] Booth RG, Strudwick G, McBride S, O’Connor S, Solano López AL (2021). How the nursing profession should adapt for a digital future. BMJ.

[11] Peine A, Neven L (2019). From intervention to co-constitution: new directions in theorizing about aging and technology. Gerontologist.

[12] Pols J (2023). Reinventing the Good Life: An Empirical Contribution to the Philosophy of Care.

[13] Stokke R (2017). “Maybe we should talk about it anyway”: a qualitative study of understanding expectations and use of an established technology innovation in caring practices. BMC Health Serv Res.

[14] Gómez DL (2015). Little arrangements that matter. Rethinking autonomy-enabling innovations for later life. Technol Forecast Soc Change.

[15] Chang F, Östlund B, Kuoppamäki S (2023). Domesticating social alarm systems in nursing homes: qualitative study of differences in the perspectives of assistant nurses. J Med Internet Res.

[16] Mol A, Moser I, Pols J (2010). Care in Practice: On Tinkering in Clinics, Homes and Farms.

[17] Schmude T, Koesten L, Möller T, Tschiatschek S (2025). Information that matters: exploring information needs of people affected by algorithmic decisions. Int J Hum Comput Stud.

[18] Labrague LJ, de Los Santos JAA, Fronda DC (2022). Factors associated with missed nursing care and nurse-assessed quality of care during the COVID-19 pandemic. J Nurs Manage.

[19] Toms G, Verity F, Orrell A (2019). Social care technologies for older people: evidence for instigating a broader and more inclusive dialogue. Technol Soc.

[20] Seibert K, Domhoff D, Bruch D (2021). Application scenarios for artificial intelligence in nursing care: rapid review. J Med Internet Res.

[21] Saab MM, Hegarty J, Murphy D, Landers M (2021). Incorporating virtual reality in nurse education: a qualitative study of nursing students’ perspectives. Nurse Educ Today.

[22] Tan SY, Taeihagh A, Tripathi A (2021). Tensions and antagonistic interactions of risks and ethics of using robotics and autonomous systems in long-term care. Technol Forecast Soc Change.

[23] Latimer J, López Gómez D (2019). Intimate entanglements: affects, more-than-human intimacies and the politics of relations in science and technology. Sociol Rev.

[24] Chang F, Kuoppamäki S, Östlund B (2022). Technology scripts in care practice: a case study of assistant nurses’ use of a social alarm system in Swedish nursing homes. Digit Health.

[25] Wang J, Fu Y, Lou V, Tan SY, Chui E (2021). A systematic review of factors influencing attitudes towards and intention to use the long-distance caregiving technologies for older adults. Int J Med Inform.

[26] Haddon L (2023). The questions are still good: domestication theory and digital media today. Digit Cult Soc.

[27] Holstein MB, Minkler M, Bernard M, Scharf T (2007). Critical Perspectives on Ageing Societies.

[28] Rockwell J (2012). From person-centered to relational care: expanding the focus in residential care facilities. J Gerontol Soc Work.

[29] Lindeman DA, Kim KK, Gladstone C, Apesoa-Varano EC (2020). Technology and caregiving: emerging interventions and directions for research. Gerontologist.

[30] Nimrod G, Edan Y (2022). Technology domestication in later life. Int J Hum–Comput Interact.

[31] Berker T, Hartmann M, Punie Y, Ward K (2005). Domestication of Media and Technology.

[32] Søraa RA, Nyvoll P, Tøndel G, Fosch-Villaronga E, Serrano JA (2021). The social dimension of domesticating technology: Interactions between older adults, caregivers, and robots in the home. Technol Forecast Soc Change.

[33] Søraa RA, Fostervold ME (2021). Social domestication of service robots: the secret lives of automated guided vehicles (AGVs) at a Norwegian hospital. Int J Hum Comput Stud.

[34] Smits M (2006). Taming monsters: the cultural domestication of new technology. Technol Soc.

[35] Östlund B, Olander E, Jonsson O, Frennert S (2015). STS-inspired design to meet the challenges of modern aging. Welfare technology as a tool to promote user driven innovations or another way to keep older users hostage?. Technol Forecast Soc Change.

[36] Martínez C, Olsson T (2021). Domestication outside of the domestic: shaping technology and child in an educational moral economy. Media Cult Soc.

[37] Song Y, Anderson RA, Corazzini KN, Wu B (2014). Staff characteristics and care in Chinese nursing homes: a systematic literature review. Int J Nurs Sci.

[38] Sun Y, Ma X, Lindtner S, He L (2023). Data work of frontline care workers: practices, problems, and opportunities in the context of data-driven long-term care. Proc ACM Hum-Comput Interact.

[39] Hsu WL, Lan K, Dong Z, Liu HL, Yang SYR, Jeang BW (2023). Importance of sensor technology in evaluation of smart monitoring system of nursing home. Sens Mater.

[40] von Gerich H, Moen H, Block LJ (2022). Artificial Intelligence -based technologies in nursing: a scoping literature review of the evidence. Int J Nurs Stud.

[41] Maanen JV (1979). Reclaiming qualitative methods for organizational research: a preface. Adm Sci Q.

[42] Hirschauer S (2007). Puttings things into words. Ethnographic description and the silence of the social. Hum Stud.

[43] Legris P, Ingham J, Collerette P (2003). Why do people use information technology? A critical review of the technology acceptance model. Inf Manage.

[44] Mannheim I, Wouters EJM, Köttl H, van Boekel LC, Brankaert R, van Zaalen Y (2023). Ageism in the discourse and practice of designing digital technology for older persons: a scoping review. Gerontologist.

[45] Joo S, Kim SH, Lee C, Kim CO, Lim YM, Jun HJ (2021). Who matters for the subjective perceptions toward gerontechnology?. Innovation Aging.

[46] Mol A (2008). The Logic of Care.

[47] Johnson GM (2021). Algorithmic bias: on the implicit biases of social technology. Synthese.

[48] Gui X, Chen Y Making healthcare infrastructure work: unpacking the infrastructuring work of individuals.

[49] Lipscomb M (2024). Routledge Handbooks.

[50] Parker SK, Grote G (2022). Automation, algorithms, and beyond: why work design matters more than ever in a digital world. Appl Psychol.

[51] Cunningham CM, Kelly GJ (2017). Epistemic practices of engineering for education. Sci Educ.

[52] World Medical Association (2025). World Medical Association Declaration of Helsinki: Ethical Principles for Medical Research Involving Human Participants. JAMA.

