# Supplementary material for: Caregivers’ Phase-Specific Roles in Integrating Digital Technologies in Chinese Nursing Homes: Qualitative Ethnographic Study
Source: J Med Internet Res. 2026 Aug 7;28:e90354. doi: 10.2196/90354 (PMC13450808; doi:10.2196/90354)
Supplement: Supplementary file 1 [file jmir-v28-e90354-s001.docx]

| **Characteristic** | **Nursing home A** | **Nursing home B** |
| --- | --- | --- |
| Location | Urban | Rural |
| Facility Size | Middle-sized | |
| Organizational Affiliation | Nationally recognized welfare organization (Demonstration facility) | |
| Technological Infrastructure | Sensor-based detection system (motion sensors, pressure mats, wearable trackers, voice alerts, centralized data platform) | |
| Caregiver Requirements | College degree or above; vocational qualifications; minimum 2 years professional experience | |
| Resident Profile | Majority aged over 85; high physical dependency; approximately 38% with cognitive impairment | Majority aged over 82; high physical dependency; approximately 42% with cognitive impairment |
