# Supplementary material for: Caregivers’ Phase-Specific Roles in Integrating Digital Technologies in Chinese Nursing Homes: Qualitative Ethnographic Study
Source: J Med Internet Res. 2026 Aug 7;28:e90354. doi: 10.2196/90354 (PMC13450808; doi:10.2196/90354)
Supplement: Supplementary file 2 [file jmir-v28-e90354-s002.docx]

**Semi-Structured Interview (SSI) Guide**

**Methodological Note on the Iterative Interview Process:**

Interviews were conducted progressively across the six-month implementation period. Initial interviews used broader, open-ended questions focusing on caregivers' everyday routines and their general encounters with the new technology. As specific adaptation strategies emerged from early observations and interviews, the guide was iteratively refined. Later interviews included more focused probes to explore emerging phenomena in depth. The questions below represent the core exploratory areas that guided these conversations.

**Section S1.** Background & daily care routines

- Could you walk me through a typical shift? What are your primary responsibilities?
- How would you describe your general approach to providing daily care for the residents?

**Section S2.** Initial encounters & training (exploring the appropriation phase)

- Can you describe the period when you first learned that the facility was introducing the new sensor-based detection system? What was your initial reaction?
- How was the system introduced and trained to the staff? How did you experience that process?
- In those early days of using the system, how did you and your colleagues make sense of what the system was supposed to do?
- Probe (if they mention evaluating the system): Can you give me a specific example of how you figured out how the system actually worked in practice?

**Section S3.** Interacting with the physical devices (exploring the objectification phase)

- How do the new devices (like the sensors, touchscreens, or wearable pendants) fit into the physical spaces of the residents' rooms?
- Can you describe how the residents have interacted with or responded to the presence of these devices?
- Have there been any instances where the physical setup or appearance of the devices was changed after the initial installation?
- Probe (if yes): Could you walk me through a specific example of what was changed, how it was done, and why?

**Section S4.** Navigating daily workflows (exploring the incorporation phase)

- How does the system and its alerts fit into your daily workflow and tasks?
- Can you walk me through exactly what happens when an alert goes off during different parts of your shift (e.g., during busy hours vs. quiet hours)?
- Can you describe a situation where the system did not behave as you expected? How did you manage the situation and ensure care continuity?
- How does the coordination regarding the system work across different shifts (e.g., between the day team and the night team)?

**Section S5.** Communication and documentation (exploring the conversion phase)

- How is the information or data generated by the system communicated to the residents' family members? Can you share a recent example of such a conversation?
- Could you describe your process for documenting the system's alerts or writing up shift logs for the management?
- How do you feel the system's data and reports represent the actual care you provide to the residents?
- Probe (if they mention adjusting reports): Can you walk me through how you decide what specific information to include or emphasize in your reports?

**Closing**

- Looking back at when the system was first installed compared to now, how has your day-to-day work changed, if at all?
- Based on your experience, what feedback would you give to the designers of this technology or the management implementing it?
- Is there anything else about your experience with this technology that you would like to share?
