# Supplementary material for: Caregivers’ Phase-Specific Roles in Integrating Digital Technologies in Chinese Nursing Homes: Qualitative Ethnographic Study
Source: J Med Internet Res. 2026 Aug 7;28:e90354. doi: 10.2196/90354 (PMC13450808; doi:10.2196/90354)
Supplement: Supplementary file 3 [file jmir-v28-e90354-s003.docx]

| **SRQR Checklist** | | | |
| --- | --- | --- | --- |
|  | **Standards for Reporting Qualitative Research (SRQR)*** | | |
|  | <http://www.equator-network.org/reporting-guidelines/srqr/> | | |
|  |  | **Item** | **Page/line no(s).** |
| **Title and abstract** | |  |  |
|  | **Title** | Concise description identifying the study as qualitative | "A Qualitative Ethnographic Study" included in title |
|  | **Abstract** | Summary including background, purpose, methods, results, conclusions | Abstract section |
|  |  |  |  |
| **Introduction** | |  |  |
|  | **Problem formulation** | Description and significance of the problem; review of relevant theory and empirical work | Introduction |
|  | **Purpose or research questio**n | Purpose of the study and specific objectives | Introduction, final paragraph |
|  |  |  |  |
| **Methods** | |  |  |
|  | **Qualitative approach and research paradigm** | Interpretivist, ethnographic approach; domestication theory as guiding framework; abductive data collection and analysis | Methods — Qualitative Approach and Research Paradigm |
|  | **Researcher characteristics and reflexivity** | First author's STS background and ten years of ethnographic experience; research team's prior assumptions; strategies to manage presuppositions; second author's role; multidisciplinary team involvement | Researcher Characteristics and Reflexivity |
|  | **Context** | Two middle-sized nursing homes in China; one urban, one rural; both operating under nationally recognized welfare organization; competitive caregiver recruitment with minimum qualifications; both facilities implementing same sensor-based detection system; both nationally recognized demonstration facilities | Methods — Study Setting and Case Selection |
|  | **Sampling strategy** | Snowball sampling beginning with caregivers who established rapport during participant observation; selection decisions made jointly by both authors; 30 frontline caregivers (15 per institution); registered nurses and managers excluded; sampling continued until meaning saturation achieved | Methods — Semi-Structured Interviews |
|  | **Ethical issues pertaining to human subjects** | Approved by Ethics Committee of Shanghai Jiao Tong University (H20240125P-R1); informed consent obtained from all participants; data anonymized through coded identifiers (P01-P30); no financial compensation provided | Methods — Ethical Considerations |
|  | **Data collection methods** | Three-stage participant observation (early deployment, embedded use, consolidation); continuous field contact maintained during inter-observation intervals via institutional group chat; 30 semi-structured interviews; document analysis of training manuals and meeting minutes; six-month total study period | Methods — Data Collection |
|  | **Data collection instruments and technologies** | Semi-structured interview guide (see Appendix); audio recording using Tencent Meeting with automated transcription; field notes; institutional documents | Methods — Semi-Structured Interviews; Appendix |
|  | **Units of study** | 30 frontline caregivers (15 urban, 15 rural); age range 23-55; work experience 2-26 years; 23 female, 7 male | Semi-Structured Interviews; Results participant table |
|  | **Data processing** | Audio-recorded interviews transcribed verbatim using Tencent Meeting automated transcription; manual verification by both authors; translated from Chinese to English using edited verbatim approach; participant anonymity maintained through coded identifiers | Methods — Semi-Structured Interviews |
|  | **Data analysis** | Abductive and iterative process; field notes and transcripts organized chronologically and assigned to domestication phases; open coding using physical thematic mapping (adhesive notes on whiteboard); multidisciplinary team resolved ambiguous phase assignments; theoretical sensitization using domestication theory; iterative refinement to develop higher-order codes; analytical memo writing throughout | Methods — Data Analysis |
|  | **Techniques to enhance trustworthiness** | Prolonged engagement (six months); continuous contact during inter-observation intervals; triangulation across three data sources; regular analytical discussions between authors; multidisciplinary team for consensus-based resolution; reflexivity through analytical memo writing; translated excerpts reviewed by both authors; member checking at informal level and formal level | Methods — Techniques to Enhance Trustworthiness |
|  |  |  |  |
| **Results/findings** | |  |  |
|  | **Synthesis and interpretation** | Main findings organized across four domestication phases; 16 caregiver roles identified at individual and collective levels; see Table 1 | Results |
|  | **Links to empirical data** | Participant quotes throughout Results with coded identifiers, gender, years of experience, and institution type (e.g., P12, F, 8 yrs, Urban) | Results — all sections |
|  |  |  |  |
| **Discussion** | |  |  |
|  | **Integration with prior work, implications, transferability, and contribution(s) to the field** | Comparison with TAM and prior domestication studies in healthcare; extension of domestication theory to institutional care; implications for technology design and policy | Discussion |
|  | **Limitations** | Two well-resourced nursing homes; findings may not generalize to under-resourced settings; caregiver heterogeneity within facilities not fully addressed; resident voices secondary; Eastern-centric sites; cultural nuances may differ | Discussion — Limitations and Future Work |
|  |  |  |  |
| **Other** | |  |  |
|  | **Conflicts of interest** | None declared | Declarations |
|  | **Funding** | Four funding grants added | Declarations |
|  |  |  |  |
|  | *The authors created the SRQR by searching the literature to identify guidelines, reporting standards, and critical appraisal criteria for qualitative research; reviewing the reference lists of retrieved sources; and contacting experts to gain feedback. The SRQR aims to improve the transparency of all aspects of qualitative research by providing clear standards for reporting qualitative research. | | |
|  |  |  |  |
|  | **The rationale should briefly discuss the justification for choosing that theory, approach, method, or technique rather than other options available, the assumptions and limitations implicit in those choices, and how those choices influence study conclusions and transferability. As appropriate, the rationale for several items might be discussed together. | | |
|  | **Reference:** |  |  |
|  | O'Brien BC, Harris IB, Beckman TJ, Reed DA, Cook DA. **Standards for reporting qualitative research: a synthesis of recommendations.** *Academic Medicine*, Vol. 89, No. 9 / Sept 2014  DOI: 10.1097/ACM.0000000000000388 | | |
